# Precisão e confiabilidade de um teste imuno-cromatográfico rápido NS1 para diagnóstico DENV-1 no ponto de atendimento e no laboratório

**DOI:** 10.1186/s12879-017-2679-z

**Published:** 2017-08-29

**Authors:** Verónica Elizabeth Mata, Sonia Regina Lambert Passos, Yara Hahr Marques Hökerberg, Guilherme Miguéis Berardinelli, Maria Angelica Borges dos Santos, Levy Vilas Boas Fukuoka, Anna Carolina Fontoura Seixas Rangel Maciel, Cintia Damasceno dos Santos Rodrigues, Aline da Silva Santos, Raquel de Vasconcellos Carvalhaes de Oliveira

**Affiliations:** 1 Av. Brasil 4036 sala 201 A, CEP 21040-361 Rio de Janeiro, RJ Brasil; 2 Rua Leopoldo Bulhões 1480 sala 601, Rio de Janeiro, RJ CEP 21040-361 Brazil; 3 Av. Brasil 4365, CEP 21040-360 Rio de Janeiro, RJ Brasil

**Keywords:** Dengue, Sensitivity and specificity, Diagnosis, Point-of-care systems, NS1, Reproducibility of results

## Abstract

**Background:**

Rapid immunochromatographic tests (ICT) for dengue non-structural protein 1 (NS1) have shown good performance for diagnosing acute-phase dengue in serum in laboratory settings, but rarely have been assessed in whole blood and at point of care (POC). This study compare the accuracy and inter- and intra-observer reliability of the NS1 Bioeasy™ ICT in whole blood at POC versus serum in the laboratory, during a DENV-1 epidemic.

**Methods:**

Cross-sectional study involving 144 adults spontaneously demanding care in an emergency department within 4 days of onset of acute febrile illness. Accuracy of NS1 Bioeasy™ ICT was compared in whole blood and serum, both at 15 and 30 min, blinded to the reference RT-PCR or NS1 ELISA. Non-dengue patients were also tested for Zika virus with RT-PCR. Reliability of whole blood and serum readings by the same or different observers was measured by simple kappa (95% CI).

**Results:**

At 15 min, sensitivity (Sn) of NS1 Bioeasy™ ICT in whole blood/POC was 76.7% (95% CI: 68.0–84.1) and specificity (Sp) was 87.0% (95% CI: 66.4–97.2). Sn in serum/laboratory was 82% (95% CI: 74.1–88.6) and Sp 100% (95% CI: 85.8–100). Positive likelihood ratio was 5.9 (95% CI: 2.0–17.0) for whole blood/POC and 19.8 (95% CI: 2.9–135.1) for serum/laboratory. Reliability of matched readings of whole blood/POC and serum/laboratory by the same observer (k = 0.83, 95% CI: 0.74–0.93) or different observers (k = 0.81, 95% CI: 0.72–0.92) was almost perfect, with higher discordant levels in the absence of dengue. Results did not differ statistically at 5%.

**Conclusions:**

NS1 Bioeasy™ ICT in DENV-1 epidemics is a potentially confirmatory test. Invalid results at 15 min should be reread at 30 min. To optimize impact of implementing ICT in the management of false-negatives it should be incorporated into an algorithm according to setting and available specimen.

**Trial registration:**

UTN U1111-1145-9451.

## Background

Dengue virus (DENV) is an international public health problem, especially serotype 1 (DENV-1), which shows the highest frequency in the world [1]. The continents with the highest incidence of dengue are America and Asia with 84% of the 390 million cases in the world per year [2]. A systematic analysis on the global burden of dengue suggested that, in 2013, disease costs worldwide may have reached U$ 8.9 billion [3].

Clinical suspicion of dengue is based on the signs and symptoms described in the World Health Organization (WHO) guidelines, i.e., nausea/vomiting, rash, aches and pains, leucopenia, any warning sign (abdominal pain or tenderness, persistent vomiting, clinical fluid accumulation, mucosal bleeding, lethargy/restlessness, liver enlargement, and hemoconcentration concurrent with rapid decrease in platelet count). However, this criterion shows low specificity due to the absence of confirmatory laboratory tests [4].

A study conducted in the United States showed that laboratory diagnostic tests assist health decisions in 60–70% of cases [5]. The World Health Organization (WHO) recommends that ideal laboratory diagnostic tests should be affordable, sensitive, specific, user-friendly, robust, rapid (can be stored at room temperature and interpreted within 30 min), equipment-free, and deliverable to those who need them (ASSURED) [6].

Rapid immunochromatographic tests (ICT) for dengue with the detection of non-structural protein 1 (NS1) meet some of the characteristics of an ASSURED test: high specificity, ranging from 82.8% [7] to 100% [8–12], simple presentations, whether in cassette or strip format, and rapid interpretation of results (15 to 30 min) [13]. However, the sensitivity showed substantial discrepancy, ranging from 55.2% to 94.3% [10, 11].

In addition, the advantages of ICT in cassette format include the possibility of performing the test in whole blood specimens rather than serum and not requiring a specific infrastructure (equipment-free). This allows their deployment in sites with limited resources, for example at point of care (POC) [7, 12–15]. The tests offer rapid results – allowing the implementation of adequate therapy and care in time to avoid possible complications and even unnecessary costs [16]. Meanwhile, ICT in strip test format requires the use of a centrifuge, reagents, storage in a refrigerator, and test tubes to perform the reaction [7–12, 17].

Dengue NS1 ICTs in cassette or strip format have been evaluated in the last decade, mainly for serotypes 1, 2, and 3, with the best performance in the first 5 days of the illness. Still, these studies mostly used serum specimens, whether from biobanks [8, 10, 11] or processed in laboratory settings [8–12]. Lack of the test’s evaluation in whole blood specimens hinders evaluation of ICT performance at POC and the recommendation of its deployment by health systems in epidemic situations [18].

The accuracy and reliability of ICTs for dengue NS1 are essential for guaranteeing quality tests. Still, few published studies on dengue have evaluated agreement between observers with different levels of training [19] or similar levels [14, 20], varying from moderate to almost perfect agreement. No studies were identified that evaluated the reliability of ICTs according to the types of specimens (whole blood versus serum) or settings.

A literature search detected only one study that evaluated duo ICT (NS1/IgM/IgG) and single NS1 using whole blood at POC, with sensitivities of 93.9% (95% CI: 88.8–96.8) and 81.6% (95% CI:74.6–87.1), and specificities of 92% (95% CI: 81.2–96.9) and 98% (95% CI: 89.5–99.7), respectively. Inter-observer reliability proved almost perfect (kappa = 1 95% 95% CI: 0.84–1) [21].

The current study thus aimed to compare the accuracy and reliability of NS1 Bioeasy™ ICT in whole blood at POC and in serum in the laboratory in adults during a DENV-1 epidemic in Rio de Janeiro State, Brazil.

## Methods

This was a prospective diagnostic cross-sectional study that evaluated accuracy of the NS1 Bioeasy™ rapid immunochromatographic test and inter- and intra-observer reliability in whole blood specimens at point of care (POC) and serum specimens in the laboratory. The study was conducted according to the Standards for Reporting Studies of Diagnostic Accuracy (STARD) [22].

The study population consisted of patients over 18 years of age with up to 4 days of acute febrile syndrome without an established diagnosis, treated consecutively and by spontaneous demand at an emergency hospital of the Resende Municipal Health Secretariat, Rio de Janeiro State, Brazil, during a dengue epidemic in March 2015.

### Data collection

The selected patients answered a questionnaire on clinical signs and symptoms associated with dengue according to WHO 2009 criteria for Dengue diagnosis [4, 23]. All of them signed the free and informed consent form.

Three blood specimens were drawn in vacuum tubes by a nurse and four medical students. Prior asepsis was performed with 70% alcohol, followed by brachial venipuncture with a 25 × 7 mm BD Vacutainer® needle. One tube with EDTA K2 was sent to the hospital laboratory for performing a complete blood count, another with heparin for ICT with whole blood at POC and the third with clotting activator and gel separator for obtaining serum. The latter was transported to the Flavivirus Laboratory, where it remained frozen at −70 °C for subsequent characterization of the specimen and ICT with serum.

The ICTs with whole blood were performed at point of care in the hospital by a nurse, and the ICTs with serum by a biologist and the same nurse (that performed ICT with blood at POC), in the Flavivirus Laboratory of the Oswaldo Cruz Institute, FIOCRUZ, the regional reference laboratory in Rio de Janeiro State. The observers were blinded to the reference test.

### Diagnosis

#### Index test

The index test used was Dengue Eden Test NS1 ICT from the Bioeasy™ company. This is a rectangular cassette with an orifice to add whole blood, serum, or plasma, and which contains an immunochromatographic membrane coated with NS1 antigen on the test line (T) and a window to view the result. When only the control line (C) is visible, the test is considered negative; if lines C and T are visible, the result is positive; and when no line or only the T line is visible, the test result is invalid [13].

The tests were performed according to manufacturer’s instructions and with readings at 15 and 30 min. Tests with invalid results at 15 min were reread at 30 min.

#### Reference test

All samples were tested using Reverse Transcription Polymerase Chain Reaction (RT-PCR) for dengue and Platelia™ Dengue NS1 Ag-ELISA (Bio-Rad™ Laboratories, France) as reference, performed according to the protocol described by Lanciotti et al. [24] and the manufacturer’s specifications, respectively [25]. Dengue cases were defined by a positive result in at least one of them and non-dengue by negative results in both. Positive specimens according to the reference test were tested by Dengue Serion ELISA classic dengue virus IgM and IgG tests (Virion/Serion, Würzburg, Germany) [26], according to manufacturers’ specifications for characterization of primary and secondary cases, respectively.

Non-dengue patients were also tested for Zika virus with RT-PCR [27] to investigate co-circulation of these viruses.

The reference tests were performed at the Flavivirus Laboratory by two biologists blinded to the index test.

### Statistical analysis

The qualitative variables were described by simple frequencies and the quantitative variables by the median with interquartile range (IQR) or minimum and maximum. Shapiro-Wilk test showed rejection of normality for the quantitative variables, indicating the use of a non-parametric test. Verification of association in the dengue and non-dengue groups used the Mann-Whitney non-parametric test and Pearson’s chi-square test for quantitative and qualitative variables, respectively.

To assess the performance of the index test, dengue NS1 ICT, in each setting using whole blood at point of care (POC) and serum in the laboratory in readings at 15′ and 30′, compared to the standard reference, the following measures of accuracy were considered: sensitivity (Sn), specificity (Sp), positive and negative predictive values (PPV, NPV), and positive and negative likelihood ratios (LR+) and (LR-) with the respective 95% confidence intervals (95% CI). Post-test probabilities were calculated for scenarios with prevalence rates of 30%, 50%, and 83.3%, using the Fagan nomogram [28].

The sensitivity and specificity of the index test in different settings were compared for using the χ^2^ McNemar test [29].

Reliability was assessed by the variability of the ICT results in whole blood/POC and serum/laboratory with readings at 15 min. Inter-observer reliability was analyzed by the readings done by the two professionals (nurse and biologist) and intra-observer reliability by the same professional (nurse), in blinded fashion. The proportions of positive agreement (Ppos) and negative agreement (Pneg) were calculated, as well as simple and prevalence-adjusted bias-adjusted kappa (PABAK) coefficients with respective 95% CI [30]. Kappa values (k) were interpreted according to the classification by Landis & Koch [31]: poor (k < 0.0), slight (0.0 < k < 0.2), fair (0.2 < k < 0.4), moderate (0.4 < k < 0.6), substantial (0.6 < k < 0.8), almost perfect (0.8 < k < 1.00), and perfect agreement (k = 1).

Statistical significance was set at *p* < 0.05.

The software packages used were R Commander 3.2.1, WinPepi 11.50, and MedCalc 15.8 [32–34].

## Results

**Fig. 1:**
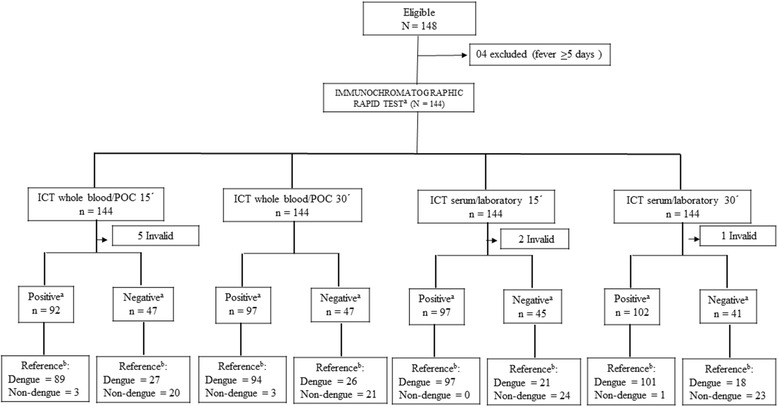
Sample selection and laboratory analysis of 144 suspected dengue cases during a DENV-1 epidemic. a: rapid immunocromatographic test (index test), b: RT-PCR or ELISA NS1 positive (reference test)

Of 148 patients recruited, four were excluded due to five or more days of fever (Fig. 1). Of the 144 patients included, 52.8% were women. Patients’ median age was 34.5 years, with higher ages in the dengue group (median: 36.1 years, ranging from 18 to 82.4) than in the non-dengue group (median: 27 years, ranging from 18.8 to 62.9), *p* = 0.019.

Patients presented a median of 1.4 days of fever (95% IQR: 1–2), and the most frequent symptoms were myalgia (91%), headache (89.6%), asthenia (83.3%), and arthralgia (73%). None of the patients evidenced clinical severity signs according to WHO 2009 criteria [4].

Prevalence of the disease was 83.3%. Of the 120 positive cases, 40.3% (*n* = 58) were primary infections. The dengue virus was characterized by RT-PCR in 105 samples and 15 exclusively by NS1 ELISA. Four samples that were negative for dengue by RT-PCR and NS1 ELISA were positive for Zika virus. One Zika Positive sample tested positive in IgM ELISA.

For all the five invalid cases on readings performed at 15 min in whole blood at POC, readings repeated at 30 min tested positive. For two serum samples at the laboratory showing invalid readings at 15 min, one turned positive at 30 min and the other remained invalid (Fig. 1).

ICT performance was similar in the two settings independently of reading times, both in the point estimate and 95% CI. Sensitivity of ICT with the 15-min reading varied from 76.7% in whole blood at point of care to 82.2% in serum in the laboratory. For the 30-min reading, sensitivity was 78.3% using whole blood at point of care and 84.9% in serum in the laboratory (Table 1). Specificity at 15-was 87% for whole blood at point of care and 100% for serum at the laboratory. For 30-min readings, specificity was 87.5% for whole blood at point of care and 95.8% for serum in the laboratory.

**Table 1:** Accuracy of Bioeasy™ rapid immunochromatographic test at point of care and in the laboratory

	Readings at 15’		Readings at 30’	
	Whole blood/POC^a^	Serum/Laboratory^b^	Whole blood/POC^a^	Serum/Laboratory^b^
Sensitivity	76.7%^c^	82.2%^c^	78.3%^c^	84.9%^c^
(95%CI)	(68.0–84.1)	(74.1–88.6)	(69.9–85.3)	(77.2–90.8)
Specificity	87.0%^d^	100.0%^d^	87.5%^d^	95.8%^d^
(95% CI)	(66.4–97.2)	(85.8–100.0)	(67.6–97.3)	(78.9–99.9)
PPV	96.7%	100.0%	96.9%	99.0%
(95%CI)	(90.8–99.3)	(96.3% - 100.0)	(91.2–99.4)	(95.4–99.8)
NPV	42.6%	53.3%	44.7%	56.1%
(95%CI)	(28.3–57.8)	(37.9–68.3)	(30.2–59.9)	(39.8–71.5)
LR+	5.9	19.8^e^	6.27	20.4
(95%CI)	(2.0–17.0)	(2.9–135.1)	(2.2–18.1)	(3.0–139.0)
LR-	0.27	0.18	0.25	0.16
(95%CI)	(0.19–0.39)	(0.12–0.26)	(0.17–0.36)	(0.10–0.24)

*POC* point of care, *95% CI* 95% confidence interval, *PPV* positive predictive value, *NPV* negative predictive value, *LR+* positive likelihood ratio, *LR-* negative likelihood ratio

^a^Performed with whole blood

^b^Performed with serum

^c^P-value <0.01

^d^P-value >0.05 of χ^2^ McNemar test

^e^LR+ calculated using 97.9% specificity

Positive predictive value was high (Table 1) in the prevalence observed in the study (83.3%). In scenarios with prevalence rates of 30% (72.4% CI: 49.3–87.7) or 50% (86%, 95% CI: 69.3–94.3), the PPV remains high. The negative predictive value would increase to 78.9% (95% CI: 72.3–84.3) with a decrease in prevalence in a scenario with 50% prevalence and to 89.7% (95% CI: 85.9–92.6) in a scenario with 30% prevalence.

The reliability between whole blood/POC and serum/laboratory was almost perfect, when evaluated both by the same observer (k = 0.83 CI: 0.74–0.93) and by different observers (k = 0.81 CI: 0.72–0.92), and remained almost perfect even when adjusted by prevalence bias. However, considering the lower limit of Kappa 95% Confidence Interval, reliability interpretation decreased from almost perfect to substantial both for inter-observer (0.73) and for intra-observer 0.75) (Table 2).

**Table 2:** Reliability at 15′ of rapid immunochromatographic test comparing whole blood/POC and serum/laboratory

	Kappa	Ppos	Pneg	PABAK
Inter-observer^a^ (*n* = 135)	0.81	94%	86.7%	0.83
(95% CI)	(0.73–0.88)	(90.6–97.3)	(79.6–93.8)	
Intra-observer^b^ (*n* = 137)	0.82	94.2%	87.6%	0.84
(95% CI)	(0.75–0.89)	(90.9–97.5)	(80.8–94.5)	

*Kappa* simple kappa, *Ppos* positive proportion, *Pneg* negative proportion, *PABAK* prevalence-adjusted bias-adjusted kappa, *95% CI* confidence interval, *n* absolute number

^a^inter-observer: nurse (blood/POC) and biologist (serum/laboratory)

^b^intra-observer: nurse (blood/POC and serum/laboratory)

## Discussion

This study assessed the Bioeasy™ rapid immunochromatographic test for DENV-1 NS1 antigen in a high-prevalence setting with adults in Rio de Janeiro State, Brazil, and found similar performance when testing was compared in whole blood/POC and serum/laboratory.

This was the first study to date comparing the performance of NS1 Bioeasy™ ICT in two scenarios, one in a laboratory setting with professionals trained to perform diagnostic tests and the other in a real-world setting (point of care). The evaluation at POC was conducted in the spontaneous treatment flow in an emergency care unit during an epidemic period, with whole blood samples drawn by different professionals. The accuracy of NS1 Bioeasy™ ICT in whole blood/POC showed sensitivity of 76.7% and specificity of 87% at the 15-minuute reading, similar to that found by Gan et al. [21] for Bioline™ ICT SD with whole blood/POC in a predominantly DENV-2 sample, i.e., Sn = 81.6%, 95% CI: 74.6–87.1 and Sp = 98%, 95% CI: 89.5–99.7.

DENV-1 ICT in serum in the laboratory showed a slight increase in accuracy, i.e., Sn = 82.2% and Sp = 100%. The literature reports variability in sensitivity according to serotype, with better performance in serotypes 1, 2, and 3, varying from 69% [7] to 81.8% [8] in DENV-1, from 62% [7] to 81.8% in DENV-2, and from 61.3% to 81.2% [8] in DENV-3, as compared to variation from 44.5% [23] to 84.8% [8] in DENV-4, so that the tests need to be improved in order to perform independently of serotype.

NS1 ICT manufacturers recommend readings at 15 or 20 min [13, 35]. However, in the current study five cases of whole blood specimens at point of care that were invalid at 15 min turned positive at 30 min, and one of the two invalid results at 15 min with serum in the laboratory turned positive at 30 min. Dussart et al. [8] and Osorio et al. [14] used ICT with serum in the laboratory and also reported improved accuracy at the 30-min reading.

The high number of false-negatives suggests that this ICT should not be used to screen for cases, as recommended even by the manufacturer [13] and Andries et al. [15]. The low number of false-positives and the high likelihood of post-testing in this study suggest that the test could be used for diagnostic confirmation. Robustness of this result is strengthened by the use of composite reference test, comprising PCR Dengue [24] and ELISA Dengue NS1 Platelia. The latter has shown very high specificity (100%) in DENV-1 in a previous study [36].

Inter and intra-observer reliability was almost perfect in this study independently of the type of specimen examined (whole blood or serum), the setting (POC or laboratory), and the level of training of the professionals that performed the test. This result corroborates Gan et al. [21].

Evidence for Zika and Dengue cross reactivity has, so far, been identified for IgM ELISA dengue but not for ELISA NS1 dengue tests [27, 37]. In our study, one of four Zika positive samples showed cross-reactivity with IgM Dengue ELISA, but none cross-react with NS1 ELISA Dengue. Increasingly acute febrile syndromes will demand investigation for several flaviviruses.

A possible limitation to the study was the high prevalence of dengue, but the post-test likelihoods were provided for different prevalence scenarios. The evaluation of a single serotype, DENV-1, may have been a limitation, but it resulted from a consecutive patient sample in an epidemic period. The comparison of whole blood/POC and serum/laboratory precludes other comparisons such as serum/POC and whole blood/laboratory, but it represents the possible routine use in emergency care departments without the necessary infrastructure for processing serum specimens during epidemics.

Considering NS1 Bioeasy™ ICT an ASSURED test [6], these recommendations may be particularly useful at sites without laboratory infrastructure or in hyperendemic scenarios, since they would offer faster turnaround of test results, thus favoring diagnosis in emergencies or remote sites [16].

In health services with some laboratory infrastructure, we recommend the use of NS1 ICT with serum in the laboratory, since it is approximately three times more positive in individuals with dengue than without (LR + = 19.8) when compared to whole blood specimens at point of care (LR + = 5.9). However, in services without laboratory infrastructure or during DEN-V 1 epidemics, we recommend screening according to WHO criteria [4] based on clinical signs followed by NS1 Bioeasy™ ICT in whole blood/POC and reading at 15 min and rereading of invalid results at 30 min.

In addition, investment to improve the performance of these tests in whole blood would allow better diagnostic confirmation, potentially optimizing the flow and efficiency of care and treatment. Training strategies for different levels of health professionals involved in performing and reading the NS1 ICT in whole blood at point of care can guarantee the reliability and adequate implementation in the clinical management of suspected cases.

## Conclusions

Bioeasy™ NS1 antigen rapid immunochromatographic test showed a good accuracy during the epidemic of DENV-1, particularly for confirmatory diagnosis. It may be useful at the point of care, with no more than a drop of whole blood, since it is rapid and simple to do by minimal trained health personnel. In this context, Bioeasy™ is relevant for the early diagnosis of dengue, particularly at sites without laboratorial infrastructure and in epidemic or hyperendemic scenarios.

## Abbreviations

ASSURED: Affordable, sensitive, specific, user-friendly, robust, rapid, equipment-free, and deliverable to those who need them
C: Control line
CI: Confidence interval
DALYs: Disability-adjusted life years
DENV: Dengue virus
DENV-1: Dengue virus serotype 1
DENV-2: Dengue virus serotype 2
DENV-3: Dengue virus serotype 3
DENV-4: Dengue virus serotype 4
EDTA: Ethylenediaminetetraacetic acid
ELISA: Enzyme-linked immunosorbent assay
ICR: Immunochromatographic rapid test
IgG: Immunoglobulin G
IgM: Immunoglobulin M
Kappa: Simple Kappa
LR−: Negative likelihood ratio
LR+: Positive likelihood ratio
NPV: Negative predictive value
NS1: Nonstructural protein-1
PABAK: Prevalence and bias adjusted kappa
POC: Point of care
PPV: Positive predictive value
RT-PCR: Reverse-transcription polymerase chain reaction
S: Sensitivity
Sp: Specificity
STARD: Standards for Reporting of Diagnostic Accuracy Study
T: Test line
WHO: World Health Organization
