# Correction to: Accuracy and reliability of an NS1 rapid immunochromatographic test for DENV-1 diagnosis at point of care and in the laboratory

**DOI:** 10.1186/s12879-017-2804-z

**Published:** 2017-11-01

**Authors:** Verónica Elizabeth Mata, Sonia Regina Lambert Passos, Yara Hahr Marques Hökerberg, Guilherme Miguéis Berardinelli, Maria Angelica Borges dos Santos, Levy Vilas Boas Fukuoka, Anna Carolina Fontoura Seixas Rangel Maciel, Cintia Damasceno dos Santos Rodrigues, Aline da Silva Santos, Raquel de Vasconcellos Carvalhaes de Oliveira

**Affiliations:** 1 Av. Brasil 4036 sala 201 A, Rio de Janeiro, RJ CEP 21040-361 Brazil; 2 Rua Leopoldo Bulhões 1480 sala 601, Rio de Janeiro, RJ CEP 21040-361 Brazil; 3 Av. Brasil 4365, Rio de Janeiro, RJ CEP 21040-360 Brazil

## Correction

An error occurred during the publication of the original article [1]. This led to several sections of the text being incorrectly converted into Portuguese:

A. The title
B. Declarations
  Ethics approval and consent to participate
  Consent for Publication
  Competing Interests
  Publisher’s Note
C. Article references

A full list of all corrections follows below:

A. **Title**: Accuracy and reliability of an NS1 rapid immunochromatographic test for DENV-1 diagnosis at point of care and in the laboratory
B. **Declarations:**

**Ethics approval and consent to participate**

The study was approved by the Institutional Review Board of the Evandro Chagas Clinical Research Institute (CAAE 0066.0.009.000–11). All patients signed the free and informed consent form.

**Consent for Publication**

Not applicable.

**Competing Interests**

The authors declare that they have no competing interests.

**Publisher’s Note**

Springer Nature remains neutral with regard to jurisdictional claims in published maps and institutional affiliations.

C. **Article references** (these refer to the references cited in the original article):
  1. Guzman MG, Halstead SB, Artsob H, Buchy P, Farrar J, Gubler DJ et al. Dengue: a continuing global threat. Nat Rev. Microbiol. 2010;8(12 Suppl):S7–16.
  2. Constenla D, Armien B, Arredondo J, Carabali M, Carrasquilla G, Castro R et al. Costing Dengue Fever Cases and Outbreaks: Recommendations from a Costing Dengue Working Group in the Americas. Value in Health Regional Issues. 2015; 8C 80–91.
  3. Shepard DS, Undurradga EA, Halasa YA, Stanaway JD. The global economic burden of dengue: a systematic analysis. Lancet Infect Dis. 2016;16(8):935–941.
  4. World Health Organization. Dengue – Guidelines for diagnosis, treatment, prevention and control. Geneva: WHO; 2009, page 11. Available in: http://www.who.int/tdr/publications/documents/dengue-diagnosis.pdf Accessed 09 April 2016.
  5. Goodman C, Faulkner E, Gould C, Karnes E, Smith A, Aguiar C et al. The Value of Diagnostics Innovation, Adoption and Diffusion into Health Care. The Lewin Group, Inc. AdvaMed. July 2005. Available in: https://dx.advamed.org/sites/dx.advamed.org/files/resource/Lewin%20Value%20of%20Diagnostics%20Report.pdf. Accessed 15 Dec 2016.
  6. Peeling RW, Mabey D. Point-of-care tests for diagnosing infections in the developing world. Clin Microbiol Infect. 2010;16(8):1062–9.
  7. Fry SR, Meyer M, Semple MG, Simmons CP, Sekaran SD, Huang JX et al. The Diagnostic Sensitivity of Dengue Rapid Test Assays Is Significantly Enhanced by Using a Combined Antigen and Antibody Testing Approach. PLoS Negl Trop Dis. 2011; 5(6): e1199.
  8. Dussart P, Petit L, Labeau B, Bremand L, Leduc A, Moua D et al. Evaluation of Two New Commercial Tests for the Diagnosis of Acute Dengue Virus Infection Using NS1 Antigen Detection in Human Serum. PLoS Negl Trop Dis. 2008; 2(8): e280.
  9. Pok KY, Lai YL, Sng J and Ng LC. Evaluation of Nonstructural 1 Antigen Assays for the Diagnosis and Surveillance of Dengue in Singapore. Vector-Borne and Zoonotic Diseases. 2010; 10(10):1009–1016.
  10. Tricou V, Vu HTT, Quynh NVN, Nguyen CW, Tran CT, Farrar J, et al., Comparison of two dengue NS1 rapid tests for sensitivity, specificity and relationship to viraemia and antibody responses. BMC Infect Dis. 2010; 10:142.
  11. Lima MdRQ, Nogueira RMR, Schatzmayr HG, Santos FBd. Comparison of Three Commercially Available Dengue NS1 Antigen Capture Assays for Acute Diagnosis of Dengue in Brazil. PLoS Negl Trop Dis. 2010; 4(7): e738.
  12. Ferraz FO, Bomfim MRQ, Totola AH, Ávila TV, Cisalpino D, Pessanha JEM et al. Evaluation of laboratory tests for dengue diagnosis in clinical specimens from consecutive patients with suspected dengue in Belo Horizonte, Brazil. Journal of Clinical Virology. 2013; 58 41–46.
  13. Dengue Eden Test Bioeasy®. Korea: Bioeasy Diagnóstica Ltda. 2015. Available in: http://www.masterdiagnostica.com.br/public/files/produtos/139421463113942146313263116009.pdf Accessed 6 May 2017.
  14. Osorio L, Ramirez M, Bonelo A, Villar LA, Parra B. Comparison of the diagnostic accuracy of commercial NS1-based diagnostic tests for early dengue infection. Virol J. 2010; 7:361.
  15. Andries AC, Duong V, Ngan C, Ong S, Huy R, Sroin KK et al. Field Evaluation and Impact on Clinical Management of a Rapid Diagnostic Kit That Detects Dengue NS1, IgM and IgG. PLoS Negl Trop Dis. 2012; 6(12): e1993.
  15. Pai NP, Vadnais C, Denkinger C, Engel N, Pai M. Point-of-Care Testing for Infectious Diseases: Diversity, Complexity, and Barriers in Low- And Middle-Income Countries. PLoS Med. 2012; 9(9): e1001306.
  17. Ty Hang V, Minh Nguyet N, The Trung D, Tricou V, Yoksan S, Dung NM et al. Diagnostic Accuracy of NS1 ELISA and Lateral Flow Rapid Tests for Dengue Sensitivity, Specificity and Relationship to Viraemia and Antibody Responses. PLoS Negl Trop Dis. 2009; 3(1): e360.
  18. Palamountain KM, Baker J, Cowan LP, Essajee S, Mazzola LT, Metzler M et al. Perspectives on Introduction and Implementation of New Point-of-Care Diagnostic Tests. J Infect Dis. 2012; 205(Suppl 2): S181–190.
  19. Martínez-Veja RA, Díaz-Quijano FA, Coronel-Ruiz C, Gómez SY, Villar-Centeno LA. Evaluación de la utilidad de la prueba rápida de casete por imunocromatografía para el diagnóstico de dengue en una región endémica colombiana. Biomédica. 2009; 29:616–24.
  20. Hunsperger EA, Yoksan S, Buchy P, Nguyem VC, Sekaran SD, Enria DA et al. Evaluation of Commercially Available Diagnostic Tests for the Detection of Dengue Virus NS1 Antigen and Anti-Dengue Virus IgM Antibody. PLoS Negl Trop Dis. 2014; 8(10):e3171.
  21. Gan VC, Tan L-K, Lye DC, Pok K-Y, Mok S-Q, Chua RC-R et al. Diagnosing Dengue at the Point-of-Care: Utility of a Rapid Combined Diagnostic Kit in Singapore. PLoS ONE. 2014; 9(3): e90037.
  22. Korevaar DA, Wang J, van Enst WA, Leeflang MM, Hooft L, Smidt N et al. Reporting diagnostic accuracy studies: some improvements after 10 years of STARD. Radiology. 2015; 274:781–9.
  23. Buonora SN, Passos SRL, do Carmo CN, Quintela FM, de Oliveira DNR, dos Santos FB, et al. Accuracy of clinical criteria and an immunochromatographic strip test for dengue diagnosis in a DENV-4 epidemic. BMC Infectious Diseases. 2016; 16:37.
  24. Lanciotti RS, Calisher CH, Gubler DJ, Chang GJ and Vorndam AV. Rapid Detection and Typing of Dengue Viruses from Clinical Samples by Using Reverse Transcriptase-Polymerase Chain Reaction. J Clin Microbiol. 1992; 30(3):545–551.
  25. Platelia™ Dengue NS1 AG®. France: Bio-Rad Laboratories, Inc.. 2008. Available in: http://www.biorad.com/webroot/web/pdf/inserts/CDG/en/72830_881149_EN.pdf Accessed 6 May 2017.
  26. Lanciotti RS, Kosoy OL, Laven JJ, Velez JO, Lambert AJ, Johnson AJ et al. Genetic and Serologic Properties of Zika Virus Associated with an Epidemic, Yap State, Micronesia, 2007. Emerg Infect Dis. 2008; 14(8):1232–1239.
  27. Serion Elisa Classic Dengue Virus IgG/IgM Virion\Serion®. Germany: Institut Virion\Serion GmbH. 2012. Available in: http://www.virion-serion.de/download/flyer/Flyer_ELISA_classic_Dengue_Virus_EN.pdf. Accessed 6 May 2017.
  28. Fagan TJ. Nomogram for Bayes theorem [letter]. N Engl J Med. 1975; 293(5):257.
  29. Trajman, A. and Luiz, R. R. McNemar χ2 test revisited: comparing sensitivity and specificity of diagnostic examinations’, Scandinavian Journal of Clinical and Laboratory Investigation. 2007. 68:1, 77–80.
  30. Byrt T, Bishop J, Carlin JB. Bias, prevalence and kappa. J Clin Epidemiol. 1993; 46(5):423–9.
  31. Landis JR, Koch GG. The measurement of observer agreement for categorical data. Biometrics. 1977; 33: 159–174.
  32. R Core Team (2015). R: A language and environment for statistical computing. R Foundation for Statistical Computing, Vienna, Austria. URL https://www.R-project.org/.
  33. Abramson, JH. WINPEPI updated: computer programs for epidemiologists, and their teaching potential. Epidemiologic Perspectives & Innovations. 2011; 8:1.
  34. MedCalc Statistical Software version 12.7.8. (MedCalc Software bvba, Ostend, Belgium; 2014. Available at https://www.medcalc.org.
  35. IVB Dengue Ag NS1 OrangeLife®. Brazil: Orangelife comércio e indústria Ltda. 2016. Available in: http://www.dmed.com.br/manuais/denguens1orangelife.pdf Accessed 07 Jan 2016.
  36. Pal S, Dauner, AL, Mitra, I, Forshey, BM, Garcia, P, Morrison, AC, Halsey, ES, Kochel, TJ, Wu SJ. Evaluation of Dengue NS1 Antigen Rapid Tests and Elisa kits using clinical samples. PLoS One. 2014; 9(11): e113411.
  37. Matheus S, Boukhari R, Labeau B, Ernault V, Bremand L, Kazanji M, Rousset D. Specificity of Dengue NS1 antigen in differential diagnosis of dengue and Zika Virus Infection. Emerg Infect Dis. 2016;22(9):1691–1692.

The Publisher apologises for this error and any confusion caused.

The original article has also been updated.
